# 4-Aminopyridine Induces Nerve Growth Factor to Improve Skin Wound Healing and Tissue Regeneration

**DOI:** 10.3390/biomedicines10071649

**Published:** 2022-07-08

**Authors:** Mashanipalya G. Jagadeeshaprasad, Prem Kumar Govindappa, Amanda M. Nelson, Mark D. Noble, John C. Elfar

**Affiliations:** 1 Center for Orthopaedic Research and Translational Science (CORTS), Department of Orthopaedics and Rehabilitation, The Pennsylvania State University College of Medicine, Hershey, PA 17033, USA; jug1031@psu.edu (M.G.J.); pmg5359@psu.edu (P.K.G.); 2 Department of Dermatology, The Pennsylvania State University College of Medicine, Hershey, PA 17033, USA; anelson@pennstatehealth.psu.edu; 3 Department of Biomedical Genetics, University of Rochester Stem Cell and Regenerative Medicine Institute, University of Rochester School of Medicine and Dentistry, Rochester, NY 14642, USA; mark_noble@urmc.rochester.edu; 4 Department of Orthopaedic Surgery, University of Arizona College of Medicine, Tucson, AZ 85721, USA

**Keywords:** 4-aminopyridine, wound healing, reinnervation, nerve growth factor, tissue regeneration

## Abstract

The discovery of ways to enhance skin wound healing is of great importance due to the frequency of skin lesions. We discovered that 4-aminopyridine (4-AP), a potassium channel blocker approved by the FDA for improving walking ability in multiple sclerosis, greatly enhances skin wound healing. Benefits included faster wound closure, restoration of normal-appearing skin architecture, and reinnervation. Hair follicle neogenesis within the healed wounds was increased, both histologically and by analysis of K15 and K17 expression. 4-AP increased levels of vimentin (fibroblasts) and alpha-smooth muscle actin (α-SMA, collagen-producing myofibroblasts) in the healed dermis. 4-AP also increased neuronal regeneration with increased numbers of axons and S100^+^ Schwann cells (SCs), and increased expression of SRY-Box Transcription Factor 10 (SOX10). Treatment also increased levels of transforming growth factor-β (TGF-β), substance P, and nerve growth factor (NGF), important promoters of wound healing. In vitro studies demonstrated that 4-AP induced nerve growth factor and enhanced proliferation and migration of human keratinocytes. Thus, 4-AP enhanced many of the key attributes of successful wound healing and offers a promising new approach to enhance skin wound healing and tissue regeneration.

## 1. Introduction

Enhancing the healing of skin wounds is an important and challenging medical problem, whether these are isolated lesions or components of larger traumatic injuries. As the major protective barrier between the sterile environment inside the body and the pathogen-rich external world, the evolutionary pressure to optimize efficient healing of skin wounds has been very high, and this process is normally effective. This long history of evolutionary selection for optimized skin wound healing raises the question of whether it is even possible to improve normal healing processes. Moreover, if this is possible, can it be done with approaches that facilitate efficient movement from the laboratory to the clinic?

One of the challenges in enhancing skin wound healing is that many different cellular processes must work in concert for effective and comprehensive repair. Along with wound closure, proliferation, migration, and/or differentiation of keratinocytes, fibroblasts, Schwann cells (SCs), and neurons, regeneration of hair follicles and other cellular changes are all important in successful, healthy wound healing [1,2,3,4]. In addition, the cells of the skin need to produce and establish a healthy microenvironment comprised of extracellular matrix and specific neuropeptides and growth factors, such as transforming growth factor-β (TGF-β) and nerve growth factor (NGF) [1,5,6,7,8], all of which are necessary to promote wound healing.

Surprisingly, several calcium channel inhibitors, including amlodipine, verapamil, diltiazem, nifedipine, and azelnidipine [9,10,11,12], promote some aspects of wound healing, raising the possibility that ion channel modulators may be useful in enhancing repair. Topical verapamil application increased the rate of wound closure, the density of fibroblasts and collagen bundles, and the volume densities of blood vessels [10]. Nifedipine and amlodipine enhanced skin tensile strength and amlodipine caused faster wound closure [11]. Thus, these compounds enhance some aspects of normal wound healing, although not all components of the healing response were investigated in these reports.

A different class of ion channel inhibitors of potential interest for skin wound healing, due to its pro-reparative effects on Schwann cells and peripheral neurons, is the potassium channel blocker 4-aminopyridine (4-AP). It has been recently shown, by genetic manipulations, that the normal function of Schwann cells is important for enabling effective skin wound repair [1]. These outcomes raise the question of whether a pharmacological approach to enhancing Schwann cell recovery might have similar benefits. 4-AP is an interesting candidate for such studies, due to its ability to enhance the recovery of both Schwann cells and neurons after peripheral nerve crush injuries [13,14,15,16]. What 4-AP would do in skin injuries, however, is unknown and not predictable. 4-AP is best studied as an inhibitor of multiple voltage-gated potassium channels [17,18], which do not appear to have been studied in the context of skin wound healing. 4-AP also affects calcium levels, both by increasing intracellular calcium levels and activating high-voltage activated calcium channels [19]. Therefore, 4-AP would be predicted to change calcium levels in ways that are opposite to what occurs with calcium channel blockers, which can inhibit calcium entry post-injury, leading to the possibility that 4-AP treatment would actually inhibit wound healing.

We now report that, in mice with full-thickness dorsal skin wounds, the systemic 4-AP treatment caused more rapid wound closure, restoration of normal epidermal thickness, tissue structure, collagen levels, and cell proliferation. Thus, the 4-AP treatment enhances many of the key attributes of successful wound healing. These findings provide strong support for the hypothesis that 4-AP treatment can enhance both tissue repair and regeneration in acute injuries. The extensive prior studies on 4-AP safety and dosing [13,14,15], and its approval for the treatment of multiple sclerosis [20], make this compound of great interest for rapid transition to clinical studies.

## 2. Materials and Methods

### 2.1. Study Design

The primary objective of this study was to investigate the possible therapeutic effect of 4-AP in enhancing skin wound healing and tissue regeneration in C57BL/6 male mice. Mouse studies were carried out following the NIH’s Guide for the Care and Use of Laboratory Animals (NIH publication No. 86–23, revised in 2011) and the animal protocol was approved by The Penn State College of Medicine Institutional Animal Care and Use Committee (IACUC No.: PROTO202001314). Mice were age-matched and randomized to treatment groups: systemic 4-AP or saline. The number of animals (*n*) needed was calculated based on the conservative use of animals for the least sensitive data type and was determined based on the desired power level greater than 80% and a required *p* < 0.05. The total we started with was 8 animals per group. The probability of mice dying in unrelated experiments was 8% and was also an 8% chance of the splint coming out (nonadherent) from the wounded mice, and we excluded such mice from the study. The selected number of animals (*n* = 5; 2 wound/animal; and total 10 wounds) used per group qualifies for the classical pre-hoc power analyses. Data were generated by microscopic analysis of immunohistochemistry, immunofluorescence on fixed skin sections, and immunoblotting of tissue and cell lysates. Five animals with 10 wounds were used in functional wound closure assessments. In the same animals, one wound tissue was used for cellular and molecular studies (*n* = 5), and the other wound for tissue protein and molecular analysis (*n* = 3). We performed full-excisional wound experiments and functional wound healing analysis on three independent cohorts of mice and each cohort contained 8 animals per group.

### 2.2. Wound Healing Assay

**Figure 1:**
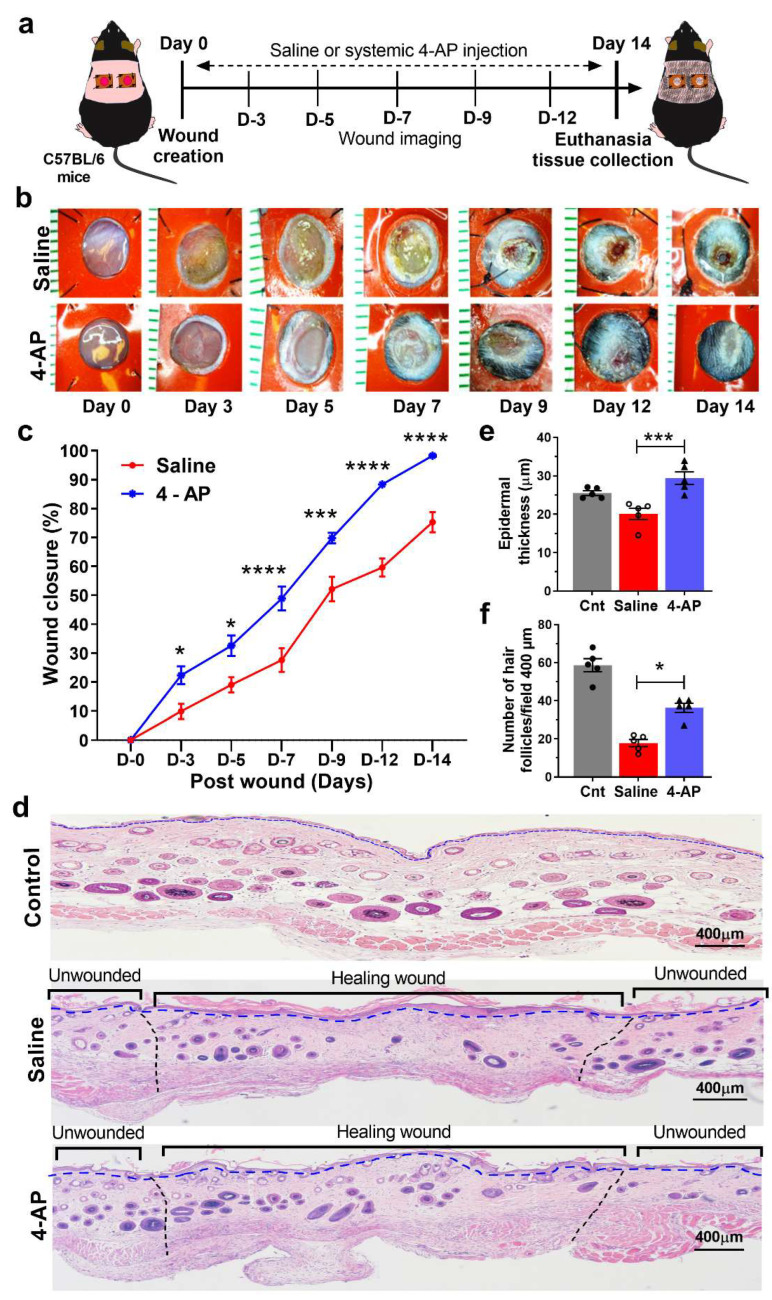
4-aminopyridine (4-AP) expedites wound closure and enhances skin regeneration. (**a**) Schematic illustration of experimental design to test the beneficial therapeutic effect of 4-AP in the C57BL/6 mouse splinted wound model. (**b**) Representative images of wound healing in control (saline-treated) and 4-AP treated mice at 0, 3, 5, 7, 9, 12, and 14 days post wounding (PWD). Scale bar, 1 mm. (**c**) Percent wound healing at each time point relative to the initial wound area in control and 4-AP-treated mice. Value represents mean ± SEM, *n =* 5 animals per group, with 2 wounds per animal, statistical significance indicated by asterisks (* = *p* between 0.01 and 0.05, *** = *p* between 0.001 and 0.0002, and **** = *p* between 0.0002 and 0.0001 vs. saline group), comparisons using two-way ANOVA (Sidak’s multiple comparisons test). (**d**) Representative images of H&E-stained sections of normal control skin and full-thickness excisional wounds of saline-control and 4-AP-treated skin tissue at PWD14. Scale bars = 400 µm. (**e**) Quantification of epidermal thickness in H&E-stained sections by ImageJ software. (**f**) Quantification of the number of de novo hair follicles within healed wounds. Each image represents 5 images from 5 different mouse wounds and data are represented as mean  ±  SEM, *n* = 5 animals per group, statistical significance indicated by asterisks (O = saline mouse data points, ∆ = 4-AP treated mouse data points,* = *p* between 0.01 and 0.05, and *** = *p* between 0.001 and 0.0002, vs. saline group).

Male C57BL/6 (10-week, 20–25 g body weight) mice were purchased from the Jackson Laboratory (Bar Harbor, ME, USA). Mice were anesthetized by intraperitoneal injection of ketamine (100 mg/kg) and xylazine (10 mg/kg) body weight, and the hair was removed by shaving and hair removal cream. Skin was disinfected using 70% ethanol and betadine before wounding. The shaved dorsal skin was folded and raised cranially and caudally at the midline to generate two, symmetrical, 5-mm-diameter wounds using a sterile punch biopsy tool (Robbin’s instrument, Houston, TX, USA, #RBP50) [1,21,22]. A 5-mm-diameter silicone ring (Grac3 Bio-labs, Bend, OR, USA, #CWS-S-0.5) was sutured (DemeTech, Miami, FL, USA #NYLON5-0) around each wound to restrict contraction. After wound creation and suturing of the silicone ring, wound sites were photographed, and the wound surface was covered with Tegaderm (3 M) sterile transparent dressing. After surgery, mice were given SR Buprenorphine (0.05 mg/kg) as postoperative analgesia. Based on the assigned treatment groups, mice received either saline or 4-AP: a saline group (vehicle control), which received 100 μL of saline, and a 4-AP group, which received 40 μg/mouse/daily 4-AP (1.6 mg/kg of 4-AP in 100 μL of saline) intraperitoneally (IP) until day 14 post-wounding. This dosage corresponds to ~40% of the mouse body surface area and is equivalent to the dosage of 20 mg/day used in treating multiple sclerosis [23] but is less than the dose that has been examined in patients with chronic spinal cord injury. Gross wound healing was monitored daily and images were captured from day 0 to day 14 (days 0, 3, 5, 7, 9, 12, and 14) post-surgery (Figure 1a). Wound areas were measured in pixels using ImageJ-1.53e software (National Institutes of Health, Bethesda, MD, USA) and normalized/corrected for each wound area with reference scales. Wound healing is expressed as a percentage with respect to day 0 wounds [1,21,22], using the following formula.

(1) $$\mathrm{Wound}\;\mathrm{healing}\;\left(\%\right)=\frac{\left(\mathrm{Area}\;\mathrm{of}\;\mathrm{orginal}\;\mathrm{wound}\;\mathrm{at}\;\mathrm{day}\;0-\;\mathrm{Area}\;\mathrm{of}\;\mathrm{wound}\;\mathrm{at}\;\mathrm{postulated}\;\mathrm{day}\right)}{\mathrm{Area}\;\mathrm{of}\;\mathrm{orginal}\;\mathrm{wound}\;\mathrm{at}\;\mathrm{day}\;0}\times 100.$$

### 2.3. Histomorphometry Analysis

Wound assessments were conducted at 14 days post-wounding using formalin-fixed (Sigma Aldrich, St Louis, MO, USA,# HT5011-CS) and paraffin-embedded tissue, serially cut into 5 μm sections on a Microtome (GMI, Ramsey, MN, USA, # Leica RM2235). Skin sections were processed for morphometric analysis using hematoxylin and eosin (H&E) (Sigma Aldrich, St Louis, MO, USA, #MHS32-1L) and immunofluorescence staining [1,21,22]. To evaluate the epidermal thickness, and wound-induced hair neogenesis (WIHN), four fields of view per sample were imaged by light microscopy (Olympus BX53, Olympus, Tokyo, Japan) at 10 and 40× magnification. Data were averaged for each mouse and then compared between 4-AP and saline-treated groups. Collagen formation, maturation, and deposition were carried out using Masson’s trichrome stain as in [24], as per manufacturer instructions (Sigma Aldrich, St Louis, MO, USA, # HT15-1KT). The Masson’s trichome stained slides were imaged by light microscopy (Olympus BX53, Olympus, Tokyo, Japan) at 20× magnification, and collagen deposition analysis was performed using ImageJ-1.53e software.

### 2.4. Immunofluorescence Staining of Tissue

Briefly, immunofluorescence analysis was performed on 5-µm-thick healed wound sections. The following primary antibodies were used: S100 antibody (Thermo Scientific Fischer-Invitrogen, # MA5-12969; 1:200), rabbit nerve growth factor receptor–p75-NTR antibody (MilliporeSigma/Sigma Aldrich, St Louis, MO, USA, # AB1554; 1:500), Chicken neurofilament Heavy-NFH antibody (Novus Biologicals, Centennial, CO, USA, # NB300-217; 1:500), mouse alpha-smooth muscle actin–α-SMA antibody (Thermo Scientific Fischer-Invitrogen, Waltham, MA, USA, # 14-9760-82; 1:200), rabbit Ki67 antibody (Cell-Signaling, Danvers, MA, USA, # 9129S; IF-1:400), chicken keratin 15 antibody (BioLegend, San Diego, CA, USA, # 833901; 1:500), rabbit K17 antibody (gift from Dr. Pierre Coulombe to AMN; 1:1000), mouse Cytokeratin14 antibody (Novus Biologicals, Centennial, CO, USA, # NBP2-34270; 1:100), mouse anti-SOX10 antibody (Santa Cruz Biotechnology, Dallas, TX, USA, # sc-365692; 1:100), rabbit vimentin antibody (Thermo Scientific Fischer, Waltham, MA, USA, # 10366-1-AP; 1:200), rabbit NGF antibody (Thermo Scientific Fischer-Invitrogen, Waltham, MA, USA, # MA5-32067; 1:100), keratin-10 (Sigma Aldrich, St Louis, MO, USA, # SAB4501656; 1:100), mouse PGP 9.5 antibody (Thermo Scientific Fischer-Invitrogen, Waltham, MA, USA, # PA5-29012; 1:200), and rat substance P antibody (Novus Biologicals, Centennial, CO, USA, # NB100-65219; 1:100). Sections were incubated with 5% BSA in 0.1% PBS-T overnight at 4 °C. Then, incubated with secondary antibodies (Thermo Scientific Fischer-Invitrogen, Waltham, MA, USA, Goat anti-Mouse IgG (H+L) or/Goat anti-Rabbit IgG (H+L) or/Goat anti-Chicken IgG (H+L) or/Goat anti-rat IgG (H+L) Highly Cross-Adsorbed Secondary Antibody, Alexa Fluor 594 /488/647, #A11008/A11032/A21449/A21247; 1:500) for 1 h at room temperature. The ProLong™ Gold Anti-fade Mountant with DAPI (Thermo Scientific Fischer-Invitrogen, Waltham, MA, USA, # P36935) was used as a nuclear counterstain. The immunofluorescence-stained sections were imaged using ZEISS Axio Observer 7-Axiocam 506 mono–Apotome.2 microscope. The image analysis and quantification were carried out using ZEN 2.6 pro (Zeiss) imaging software or ImageJ-1.53e software.

### 2.5. Human Primary Cell Culture Experiments

Foreskin collection and preparation: The human foreskin was rinsed gently with 1X-PBS (ScienCell Research, Carlsbad, CA, USA, #0303) containing an antibiotic. The hypodermis and blood vessels were removed. Subsequently, the skin was cut into 1–2 mm pieces and then placed in DMEM medium (ScienCell Research, Carlsbad, CA, USA, #09221) with dispase-I (Sigma Aldrich, St Louis, MO, USA, # D46693) at 4 °C for 12–18 h. After dispase-I treatment, the epidermis was separated from the dermis [25,26,27].

Keratinocyte isolation, culture conditions, and characterization: The isolated epidermis was placed in a petri dish containing HBSS buffer (Lonza, Basel, Switzerland, # CC-5022) for 10 min at room temperature, then treated with trypsin (Lonza, Basel, Switzerland, # CC-5012) at 37 °C until the epidermis became loose and the medium became cloudy due to keratinocyte release. The cloudy medium was collected and trypsin activity was neutralized using fetal bovine serum (FBS, Thermo Scientific Fischer, Waltham, MA, USA, # 10082147) in a 1:1 ratio. The epidermis and suspended keratinocytes were centrifuged at 1500 rpm for 5 min. The pellet was resuspended in KGM-GOLD keratinocyte medium (Lonza KGM gold and supplements, # 00192151 and 00192152) [25,26,28]. The isolated foreskin was cultured at 37 °C in a 5% CO_2_ incubator for 1–2 days to allow keratinocytes to adhere. Adherent keratinocytes were maintained in KGM-Gold medium until cells reached about 80% confluent. Indirect immunofluorescence analysis was used to identify and characterize keratinocytes that were processed in the same experimental session. An equal number of passage 1 cells were seeded on chamber slides. The cells were grown in a respective complete medium. Cells then were fixed with 4% paraformaldehyde followed by 0.1% triton X-100 and stained with primary antibodies used against, cytokeratin-14, and keratin-10 in 5% BSA-containing PBS. After washing, cells were incubated with respective secondary antibodies and washed with PBS after incubation. After DAPI labeling, the chamber glass slides were mounted using Prolong Gold anti-fade mounting medium and then covered with glass coverslips.

### 2.6. Cell Viability Assay with 4-Aminopyridine

The keratinocytes were cultured in 96-well plates for 18 h. Cells were placed in minimal media (no serum or growth factors) for 4 h before 4-AP treatment. Cells were treated with 4-AP (at concentrations ranging from 1 to 10,000 µM) inappropriate cell culture medium for 24 h. The cell viability following 4-AP treatment was assayed by MTT assay according to the manufacturer’s protocols (Roche, cell proliferation kit I (MTT), # 11465007001). The percentage of live keratinocytes was determined using the following formula.

$$\mathrm{Cell}\;\mathrm{viability}\;\left(\%\right)=\frac{\left(\mathrm{OD}\;\mathrm{of}\;4-\mathrm{AP}\;\mathrm{treated}\;\mathrm{cells}\;\mathrm{at}\;\mathrm{particular}\;\mathrm{concentration}\;-\;\mathrm{OD}\;\mathrm{of}\;\mathrm{medium}\right)}{\mathrm{OD}\;\mathrm{of}\;\mathrm{control}\;\mathrm{cells}\;\left(\mathrm{No}\;\mathrm{treatment}\right)-\;\mathrm{OD}\;\mathrm{of}\;\mathrm{medium}}\times 100.$$

### 2.7. Cell Scratch Wound Healing Migration Assay

Keratinocytes (7 × 10^4^ cells/well) were seeded on tissue culture dishes pre-coated either with collagen-I (corning life science, Durham, NC, USA, # 354236) on 96-well ImageLock microplates for 6 h (Incucyte-sartorius plate, Goettingen, Germany, # 4379). For drug treatment, cells were pretreated with 4-AP for 18 h before performing the wound scratch assay. Next, wound scratches were created using the IncuCyte automated system (Incucyte-sartorius-Essen BioScience, Goettingen, Germany) [26,29,30]. After scratching, the cells were washed with PBS, and the KC media was added with or without 1 mM of 4-AP. The plate was incubated in the IncuCyte™ automated imaging system and wound healing and cell migration were monitored by time-lapse photography capturing images every hour from 0 to 24 h. The relative area of wound size and cell migration at each time point was analyzed using the IncuCyte™ Scratch Wound Cell Migration Software Module (Essen BioScience) and the percent of wound healing was calculated from the area measured after scratching relative to the basal area as expressed in pixels.

### 2.8. Tissue Protein Isolation and Western Blot Analysis

For protein isolation, the harvested skin tissue was flash frozen immediately. The frozen skin tissue was ground to a fine powder using a liquid nitrogen mortar. The harvested cells and/or tissue powder were dissolved in RIPA buffer containing Halt™ Phosphatase (Thermo Scientific Fischer, Waltham, MA, USA, # 78420) and Protease Inhibitor Cocktail (Sigma Aldrich, St Louis, MO, USA, Roche complete tablets mini EASYpack, # 04694124001). Tissue and cell debris were removed by centrifugation at 14,000 rpm for 30 min at 4 °C. The supernatants were collected and the total protein concentration was determined by BCA protein assays (Thermo Scientific Fischer, Pierce^TM^, Waltham, MA, USA, # 23225). The proteins (20–30 μg) of the tissue protein samples were subjected to 12% sodium dodecyl sulfate-polyacrylamide gel electrophoresis (Bio-Rad Laboratories, Hercules, CA, USA, mini-PROTEAN TGX Gels, # 4561044) and transferred to polyvinylidene fluoride (PVDF) membranes. After the membranes were blocked with 5% non-fat milk in 1X TBS-T for 1 h, they were incubated with the appropriate primary antibodies (rabbit nerve growth factor receptor–p75-NTR antibody (Sigma Aldrich, St Louis, MO, USA, # AB1554; 1:500), mouse alpha-smooth muscle actin–α-SMA antibody (Invitrogen, # 14-9760-82; 1:500), rabbit TGF-β antibody (Cell-Signaling, Danvers, MA, USA, #3711S, 1:500), mouse anti-SOX10 antibody (Santa Cruz Biotechnology, Dallas, TX, USA, # sc-365692; 1:100), rabbit NGF antibody (Thermo Scientific Fischer-Invitrogen, Waltham, MA, USA, # MA5-32067; 1:1000), Mouse GAPDH antibody (Thermo Scientific Fischer, Waltham, MA, USA, #MA5-15738; 1:1000) at 4 °C overnight, then incubated with HRP-conjugated secondary antibodies (dilution, 1:3000) for 1 h. Immunoreactivity was then detected using chemiluminescent substrate (Thermo Scientific Fischer, Waltham, MA, USA, SuperSignal™ West Pico PLUS, # 34577). The intensities of the bands were quantified using Gel-imaging software ((Bio-Rad Laboratories, Hercules, CA, USA, Image Lab 6.1). The quantified band intensities were normalized using GAPDH and expressed either as normalized intensity or as ratios concerning saline-treated mice.

### 2.9. Statistical Analysis

The number of animals per group was determined by pre-hoc power analysis of preliminary data by a qualified statistician to achieve at least 80% power for the primary outcome in this study. Animals were randomly assigned to either saline treatment group. All results are presented as mean ± standard error of the mean (SEM). Data were analyzed using two-tailed Sidak’s for wound healing functional analysis multiple time-point comparisons and unpaired data from different experiments by one-way ANOVA followed by unpaired *t*-test and nonparametric test after confirmation of normally distributed data. Statistical analysis was performed using the GraphPad PRISM 9.2.0(332) (GraphPad, La Jolla, CA, USA) and the star indicates statistical significance of * = *p* between 0.01 and 0.05, ** = *p* between 0.01 and 0.001, *** = *p* between 0.001 and 0.0002, and **** = *p* between 0.0002 and 0.0001 versus saline considered as significant.

## 3. Results

### 3.1. 4-Aminopyridine (4-AP) Accelerates Wound Closure and Enhances Skin Regeneration

Systemic treatment with 4-AP accelerated full-thickness skin wound closure. We created 5-mm-diameter full-thickness dorsal excisional wounds in 10-wk-old male C57BL/6 mice [21]; mice were then randomized and treated with either saline or systemic 4-AP [31] daily for 14 days (Figure 1a). Wounds were splinted with silicone rings to prevent wound contraction and were monitored by digital imaging for morphometry, percentage of wound healing, and tissue regeneration on days 3, 5, 7, 9, 12, and 14 post wound (PWD) (Figure 1a).

We found that the extent of wound closure in 4-AP-treated mice was more than twice that of saline-treated mice at PWD3 (Figure 1b,c). Significant differences in wound closure were noted at every time point examined, including PWD14 (*p* < 0.0001). At this point, 4-AP-treated mice had complete wound closure, while open wounds were still present in saline-treated mice (Figure 1b,c). To confirm that saline treated mice had complete wound closure, we followed a separate cohort of mice to complete wound closure. Saline-treated mice had complete wound closure at PWD18, four days after complete wound closure in 4-AP mice (data not shown).

Histomorphometry analysis on PWD14 skin sections revealed that 4-AP treatment increased epidermal thickness compared with saline-treated mice, with the resulting epidermal thickness in 4-AP treated mice being restored to that of uninjured skin (Figure 1d,e) [1,22].

4-AP treated mice also showed a significant increase in wound-induced hair neogenesis (WIHN) when compared with saline-treated mice (Figure 1d,f), a feature of successful skin regeneration [32,33]. The phenomenon of WIHN was described and characterized by Ito et al., in 2007, and showed that after full-thickness wounding in mice, regenerated hair follicles within the healed wound establish a stem cell population, express hair follicle-differentiation markers, produce a functional hair shaft, and successfully transition through all phases of the hair cycle [32]. 4-AP-treated mice exhibited a 1.8-fold increase in the number of hair follicles compared to saline-treated mice (Figure 1f), highlighting that 4-AP enhances skin regeneration.

### 3.2. 4-AP Increases Keratinocyte Number and Epithelial Stem-Cell Markers in Healed Wounds

**Figure 2:**
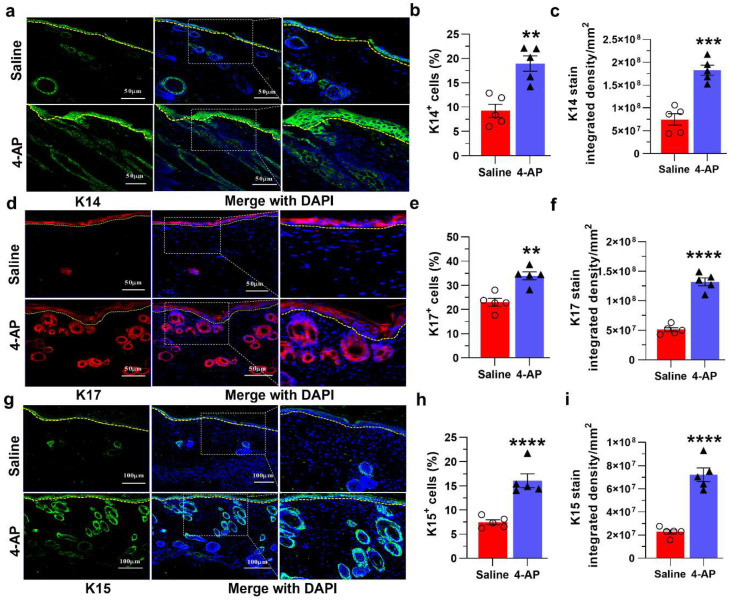
4-AP increases keratinocyte number and epithelial stem-cell markers in healing wounds. (**a**) Representative images of Keratin 14 protein expression by immunofluorescence in healed epidermis. K14 (green); DAPI (blue) denotes nucleus and dashed line denotes epidermal/dermal border. (**b**,**c**) Percent of K14^+^ cells and K14 protein integrated density in saline-control and 4-AP-treated skin wounds at day 14. Scale bars = 50 μm. Data represents 20 images from 5 different mouse wounds and are shown as mean  ±  SEM, *n* = 5 animals per group, statistical significance indicated by asterisks (O = saline mouse data points, ∆ = 4-AP treated mouse data points, ** = *p* between 0.01 and 0.001, and *** = *p* between 0.001 and 0.0002 vs. saline). (**d**) Representative images of keratin 17 protein expression by immunofluorescence in saline and 4-AP-treated skin wounds. K17 (red); DAPI (blue) denotes nucleus and dashed line denotes epidermal/dermal border. (**e**,**f**) Percent of K17^+^ cells and K17 protein integrated density in control and 4-AP treated skin wounds at day 14; scale bars = 50 μm. Data represents 20 images from 5 different mouse wounds and are shown as mean  ±  SEM, *n* = 5 animals per group, statistical significance indicated by asterisks (O = saline mouse data points, ∆ = 4-AP treated mouse data points, ** = *p* between 0.01 and 0.001, and **** = *p* between 0.0002 and 0.0001 vs. saline). (**g**) Representative images of keratin 17 protein expression by immunofluorescence in saline and 4-AP-treated skin wounds. K15 (green); DAPI (blue) denotes nucleus and dashed line denotes epidermal/dermal border. (**h**,**i**) Percent of K15^+^ cells and K15 protein integrated density in saline control and 4-AP-treated skin wounds at day 14. Scale bars =100 μm. Data represents 20 images from 5 different mouse wounds and are shown as mean  ±  SEM, *n* = 5 animals per group, statistical significance indicated by asterisks (O = saline mouse data points, ∆ = 4-AP treated mouse data points, **** = *p* between 0.0002 and 0.0001 vs. saline).

Given the thickened epidermis in healed wounds with 4-AP (Figure 1d,e), we investigated whether this was associated with increased numbers of keratinocytes and/or altered epidermal differentiation. We observed a 2-fold increase in the number of keratin-14 positive (K14^+^) keratinocytes in the epidermis and the de novo hair follicles of 4-AP-treated mice (Figure 2a) compared with saline-treated mice (Figure 2a–c). In contrast, the expression of K10, a marker of epidermal differentiation, was not impacted by 4-AP treatment at this time point (Supplementary Figure S1a,b) [34].

In agreement with the increased numbers of hair follicles noted by histology (Figure 1d,f), 4-AP increased the number of K17^+^ and K15^+^ cells and the overall expression of these proteins further corroborating the increase in hair follicle numbers [32,35,36]. We observed a 1.5-fold increase in the percentage of K17^+^ cells, and in K17 protein expression (Figure 2d–f). Similarly, K15^+^ cells and protein expression were increased with 4-AP treatment (Figure 2g–i).

K14 and K17 expression also increased in the overlying epidermis in both saline and 4-AP-treated mice compared to uninjured skin, which is consistent with their known upregulation in response to wounding (Figure 2a,d) [37].

### 3.3. The 4-AP Treatment Promotes Increases in Fibroblasts, Myofibroblasts and Transforming Growth Factor-β (TGF-β)

Fibroblast migration and maturation contribute to the contraction, granulation, and proliferation phases of wound healing. A key marker of fibroblast differentiation is α-smooth muscle actin (α-SMA) which signifies fibroblast differentiation into collagen-producing myofibroblasts [1,3,22]. We, therefore, investigated the effects of 4-AP treatment on fibroblasts and the expression of a known regulator of fibroblast differentiation, TGF-β.

**Figure 3:**
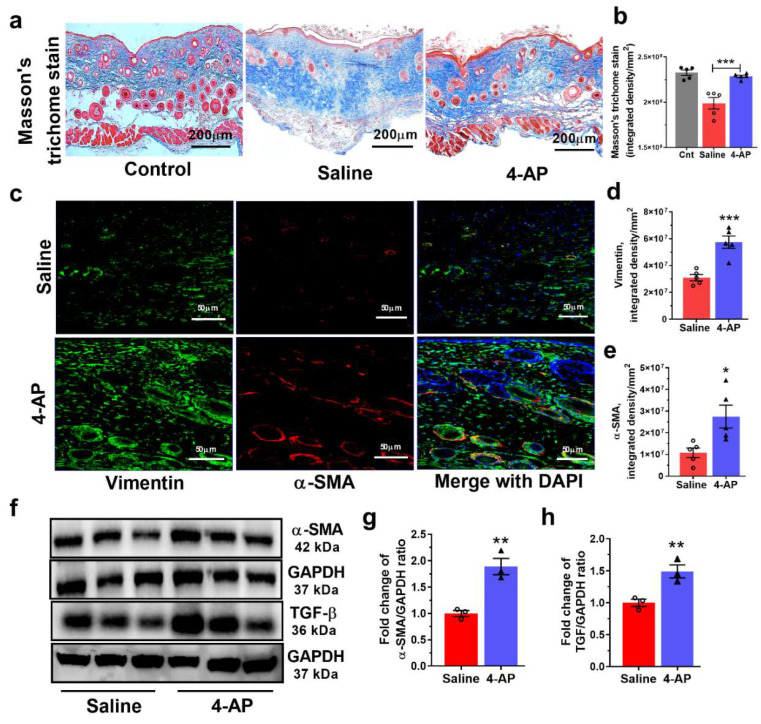
4-AP treatment increases fibroblast and myofibroblast numbers and levels of transforming growth factor-β. (**a**) Representative Masson’s trichrome stained images of healing control skin and full-thickness excisional wound of saline-control and 4-AP-treated skin tissue at day 14 (PWD14). Scale bars = 200 μm. (**b**) Collagen density quantified as average blue pixel density per area in wound healing tissue harvested on day 14 (PWD 14). Data represents 10 images from 5 different mouse wounds and are shown as mean  ±  SEM, *n* = 5 animals per group, statistical significance indicated by asterisks (O = saline mouse data points, ∆ = 4-AP treated mouse data points, *** = *p* between 0.001 and 0.0002 vs. saline). (**c**) Co-immunofluorescence staining of vimentin (green), α-SMA (red), and nuclear stain (DAPI-blue) in saline control and 4-AP-treated skin wound sections at day 14. Scale bars = 50 μm. (**d**,**e**) Quantification of vimentin and α-SMA protein staining intensity (integrated density). Data represent 20 images from 5 different mouse wounds and are shown as mean  ±  SEM, *n* = 5 animals per group, statistical significance indicated by asterisks (O = saline mouse data points, ∆ = 4-AP treated mouse data points, * = *p* between 0.01 and 0.05, and *** = *p* between 0.001 and 0.0002 vs. saline). (**f**) Representative Western blots of α-SMA and TGF-β levels. (**g**,**h**) Quantitation of α-SMA and TGF-β levels to normalized to GAPDH (fold change mean ± SEM, *n = 3* animals per group, statistical significance indicated by asterisks (O = saline mouse data points, ∆ = 4-AP treated mouse data points, ** = *p* between 0.01 and 0.001vs. saline)).

To test whether 4-AP treatment altered fibroblast maturation during wound healing, we first performed Masson’s Trichrome staining to examine collagen deposition in the healing wound (Figure 3). This staining revealed elevated collagen deposition in 4-AP-treated mice compared to saline-treated mice (Figure 3a,b), with collagen levels like those seen in normal tissue. This staining also revealed a tissue structure and collagen deposition pattern very similar to that seen in normal tissue.

4-AP treatment also increased the expression of fibroblast proteins, vimentin, and α-smooth muscle actin (α-SMA). Immunofluorescence analysis revealed more vimentin^+^ fibroblasts and elevated vimentin levels in wound tissue from 4-AP-treated mice than saline-treated mice (Figure 3c,d), which was consistent with Western blot (WB) analysis for α-SMA expression (Figure 3e,f). We also observed increases in α-SMA, which signifies fibroblast differentiation into collagen-producing myofibroblasts [1,3]. Increases were also seen in α-SMA protein (Figure 3c,e–g).

TGF-β plays an important role in promoting myofibroblast differentiation [1,3,8], and we found significant increases in TGF-β protein expression with 4-AP treatment compared to saline treatment (Figure 3f,h).

### 3.4. 4-AP Promotes Reinnervation and Neuropeptide Expression

Normal skin wound healing is also associated with increases in cell division and increases in non-dividing neurons. 4-AP treatment caused increases in both measures.

**Figure 4:**
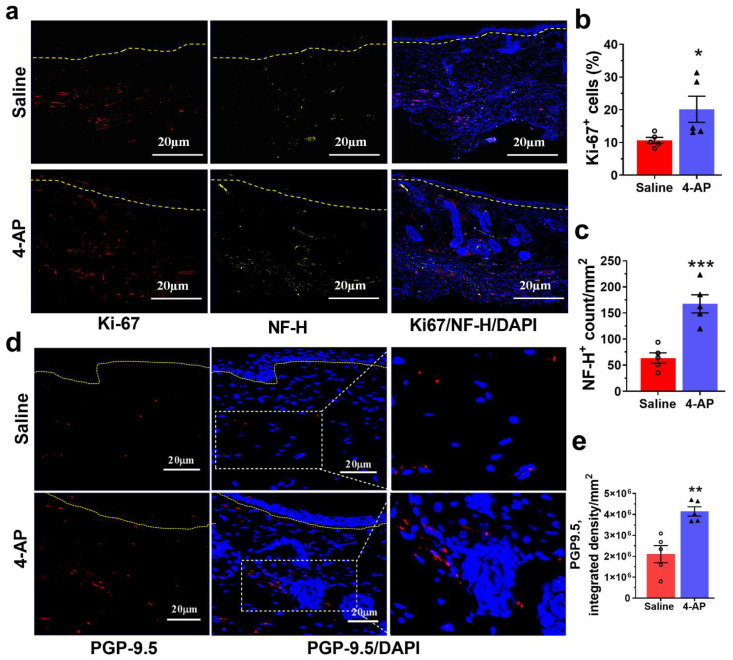
4-AP promotes division reinnervation and enhanced PGP-9.5 expression. (**a**) Representative image of co-immunostaining for Ki-67^+^ cells (red) and NF-H^+^ cells (yellow) surrounding de novo hair follicles in the healed wound at day 14 and dashed line denotes the epidermal/dermal border. Scale bars = 20 μm. (**b**,**c**) Ki67^+^ and NF-H^+^ cells were quantified. Data represents 20 images from 5 different mouse wounds and are shown as mean  ±  SEM, *n* = 5 animals per group, statistical significance indicated by asterisks (O = saline mouse data points, ∆ = 4-AP treated mouse data points,* = *p* between 0.01 and 0.05, and *** = *p* between 0.001 and 0.0002 vs. saline). (**d**) Representative images of PGP-9.5 protein expression by immunofluorescence staining of healed wounds. PGP-9.5 (red) and nuclear stain DAPI (blue) and the dashed line denotes the epidermal/dermal border. Scale bars = 20 μm. (**e**) Quantification (integrated density) of PGP-9.5 protein expression. Data represents 20 images from 5 different mouse wounds and are shown as mean  ±  SEM, *n* = 5 animals per group, statistical significance indicated by asterisks (O = saline mouse data points, ∆ = 4-AP treated mouse data points, ** = *p* between 0.01 and 0.001 vs. saline).

Expression of the proliferation marker, Ki-67, was significantly increased in mice treated with 4-AP compared with saline-treated controls. The proportion of Ki-67^+^ cells within hair follicles and epidermis was increased 2.0-fold (Figure 4a,b).

The number of neurons in the skin of 4-AP-treated mice also was increased over that seen in saline-treated animals. The neuronal number was determined by staining with antibodies against high molecular weight neurofilament protein (NF-H) [38]. NF-H axonal counts were increased 2.5-fold in 4-AP-treated mice compared with saline-treated controls (Figure 4a,c).

We also found that NF-H-stained axons in the 4-AP-treated mice were more often encountered in direct association with Ki-67^+^ hair follicles (Figure 4a) than in saline-treated controls, in agreement with observations that hair follicles are associated with sympathetically innervated *arrector-pili-muscles* [37]. Thus, the association between nerve function and hair follicle stem cells under the influence of 4-AP supports reinnervation as a possible factor in the formation of de novo hair follicles during wound healing.

Another example of the ability of 4-AP to restore aspects of skin structure like that seen in uninjured tissue was revealed by staining for protein gene product 9.5 (PGP-9.5), a neuronal peptide associated with wound healing [39]. Fourteen days post wounding, PGP-9.5^+^ nerve fibers in the healed wounds were twice as abundant in 4-AP-treated mice, as reflected by increased amounts of PGP-9.5, as compared with saline-treated mice (Figure 4d,e). The levels of PGP-9.5 in 4-AP-treated mice were not significantly different from those seen in uninjured skin tissue (Supplementary Figure S1c,d). This suggests that 4-AP significantly increased the expression of PGP-9.5 during wound healing and that 4-AP likely enhances skin reinnervation.

### 3.5. 4-AP Increases the Numbers of Schwann Cells (SC) and the Expression of Markers of an Early Differentiation State

**Figure 5:**
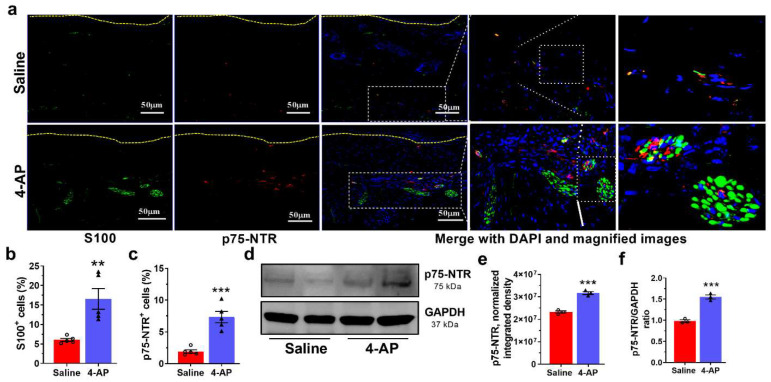
4-AP increases the number of Schwann cells and the expression of markers of an early SC differentiation state. (**a**) Representative image of co-immunostaining of S100 (green) and p75-NTR (red) in wound sections and dashed line denotes epidermal/dermal border. Scale bars = 50 μm. (**b**,**c**) Quantification of S100^+^ and p75-NTR^+^ expressing cells in healed wounds. Data represents 20 images from 5 different mouse wounds and are shown as mean  ±  SEM, *n* = 5 animals per group, statistical significance indicated by asterisks (O = saline mouse data points, ∆ = 4-AP treated mouse data points, ** = *p* between 0.01 and 0.001, and *** = *p* between 0.001 and 0.0002 vs. saline). (**d**) Representative image of Western blot of p75-NTR and GAPDH. (**e**,**f**) Normalized integrated densities and the ratio of p75-NTR protein expression represented as mean ± SEM, *n* = 5 animals per group, statistical significance indicated by asterisks (O = saline mouse data points, ∆ = 4-AP treated mouse data points, *** = *p* between 0.001 and 0.0002 vs. saline).

Schwann cells (SC) are critical players in wound healing and are associated with axons around hair follicles in the wound bed. In the setting of injury, SCs de-differentiate to a non-myelinating state and begin to secrete neurotrophins such as NGF, a state marked by expression of p75-NTR [40]. We found that the number of SCs was significantly increased in the wounds of 4-AP-treated mice (Figure 5a,b). Analysis of expression of S100, a pan-SC marker, identified SCs within both the hypodermis and dermis of the healed wounds (Figure 5a). The number of SCs was 3-fold greater in 4-AP-treated mice than in saline-treated controls (Figure 5a,b). SCs were preferentially located around nerve bundles, as predicted by the known affiliation of SCs with nerve cells. 4-AP treatment also increased the expression of p75-NTR (Figure 5a,c–f), which is thought to be expressed in S100^+^ cells as a marker of de-differentiation [1,41].

We also found elevated expression of SOX10 and NGF in 4-AP-treated mice. SOX10 is required for myelin production in SCs and elevated SOX10 expression promotes the conversion of mesenchymal cells into p75-NTR expressing neural crest stem cells (NCSC) [1,42,43,44]. Conversely, depletion of SOX10 expression significantly delays wound healing and tissue regeneration [1]. NGF plays a significant role in the wound healing process by inducing nerve sprouting from injured nerve endings [5,7,29,45,46]. NGF also promotes keratinocyte proliferation and migration of dermal fibroblasts [6,47]. NGF also acts on non-neuronal cells to sensitize them to substance-P, which in turn further stimulates more NGF secretion, ensuring that keratinocytes can elaborate and respond to neuronal factors along with neurons [48,49].

**Figure 6:**
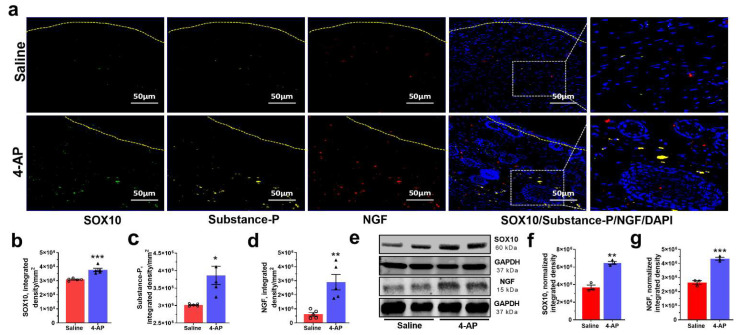
4-AP enhanced expression of transcription factors, neurotrophic factors, and neuropeptides associated with reinnervation. (**a**) Representative image of triple co-immunostaining of wound skin for the transcription factor SOX10 (green), neuropeptide substance-P (SP—yellow), nerve growth factor (NGF—red), and nuclear stain DAPI (blue) and dashed line denotes epidermal/dermal border. Scale bars = 50 μm. (**b**–**d**) Quantification of SOX10, substance-P, and NGF expressing cells in healed wounds. Data represents 20 images from 5 different mouse wounds and are shown as mean  ±  SEM, *n* = 5 animals per group, statistical significance indicated by asterisks (O = saline mouse data points, ∆ = 4-AP treated mouse data points, * = *p* between 0.01 and 0.05, ** = *p* between 0.01 and 0.001, and *** = *p* between 0.001 and 0.0002 vs. saline). (**e**) Representative image of Western blot of SOX10, NGF, and GAPDH. (**f**,**g**) Normalized integrated densities for SOX10 and NGF immunoblot. Mean ± SEM, *n = 3* animals per group, statistical significance indicated by asterisks (O = saline mouse data points, ∆ = 4-AP treated mouse data points, ** = *p* between 0.01 and 0.001, and *** = *p* between 0.001 and 0.0002 vs. saline).

We found significantly increased SOX10 protein expression by both immunofluorescence and Western blot analysis (Figure 6a,b,e,f). Substance-P and NGF protein expression was also increased in healed wounds from 4-AP-treated mice compared to saline-treated mice (Figure 6a,c–g). These factors, which are known to be associated with both nerve regeneration in the wound bed and accelerated healing [1,5,6,7,29,45,49], were increased within the wound with 4-AP treatment.

### 3.6. 4-AP Effectively Stimulates Proliferation and Migration in Primary Cultures of Human Skin-Derived Primary Cells In Vitro

We next found that the effects of 4-AP on keratinocytes and SCs in vitro were similar to outcomes observed in vivo, suggesting that 4-AP may act directly on these cell types. In these experiments, we cultured primary, normal human epidermal keratinocytes (NHEKs) in the presence or absence of 4-AP [25,26].

**Figure 7:**
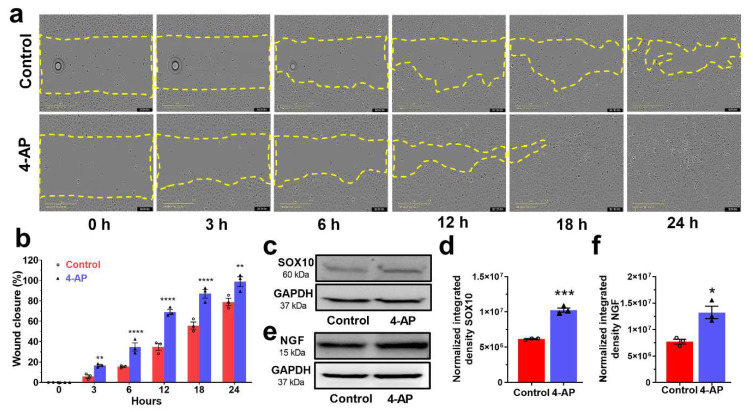
4-AP accelerates in vitro keratinocyte wound closure. (**a**) Representative images of in-vitro keratinocyte scratch assays with 4-AP and vehicle control at indicated time points. The yellow lines indicate the wound borders at the beginning of the assay and were recorded every hour until 24 h. Scale bar =100 µm. (**b**) The relative percentage of wound closure was calculated as the ratio of the remaining wound gap at the given time point compared to time 0. Each image represents 9 images from 3 biological replicates and data represented as mean  ±  SEM, *n = 3* biological replicates per group, statistical significance indicated by asterisks (O = saline mouse data points, ∆ = 4-AP treated mouse data points, ** = *p* between 0.01 and 0.001, and **** = *p* between 0.0002 and 0.0001 vs. control). (**c**–**f**) A representative Western blot and normalized integrated densities for SOX10 and NGF. Mean ± SEM, *n = 3* biological replicates per group, statistical significance indicated by asterisks (O = saline mouse data, ∆ = 4-AP treated mouse data, * = *p* between 0.01 and 0.05, and *** = *p* between 0.001 and 0.0002 vs. control).

Following initial isolation from the human foreskin, we confirmed the purity and identity of keratinocytes by immunohistochemistry using characteristic protein markers (Supplementary Figure S2a). There was a modest decrease in cell viability at higher 4-AP concentrations (>10 mM), but no obvious impact on cell viability at 2 mM concentrations of 4-AP (Supplementary Figure S2b) [38]. To determine the effect of 4-AP on wound healing in vitro, automated wound scratch assays were performed [29,38] on confluent monolayers of keratinocytes with and without 1 mM 4-AP, a standard 4-AP dose used in studies in vitro [38]. 4-AP exposure accelerated scratch closure and keratinocyte migration (Figure 7a,b; and Supplementary Movies S1–S2) within 3 h, with complete scratch closure occurring at 18 h. In contrast, control cultures not exposed to 4-AP closed at 32 h (Supplementary Movies S1 and S2).

SOX10 and NGF expression in 4-AP treated keratinocytes were both increased (Figure 7c–f), suggesting that 4-AP promotes a more proliferative, stem-like phenotype in keratinocytes that contributes to accelerated scratch closure [29,42].

## 4. Discussion

We found that, in mice with dorsal skin wounds, the systemic 4-AP treatment caused more rapid wound closure, restoration of normal epidermal thickness, tissue structure, and collagen levels, and increased vascularization and cell proliferation. Hair follicle numbers were also increased in 4-AP-treated mice, as determined histologically and by analysis of K15 and K17 expression, as were numbers of K14^+^ keratinocytes. Levels of vimentin (a marker of fibroblasts) and α-SMA (a marker of collagen-producing myofibroblasts) were increased, as were the numbers of α-SMA^+^ cells. 4-AP treatment also increased the numbers of axons and S100^+^ Schwann cells and increased the expression of SOX10. Levels of several factors involved in skin repair also were increased (i.e., TGF-β, NGF, and Substance P). Thus, 4-AP enhanced many of the key attributes of successful wound healing. As an already approved therapeutic agent, 4-AP appears to offer a promising new approach to wound healing and skin regeneration.

Enhancing normal skin wound healing is a difficult challenge because of how effective this process already is. Expediting and enhancing the wound healing process, however, could help in restoring the skin barrier even more quickly and in mitigating infection complications. The many millions of years of evolutionary optimization of this process have resulted in a complex orchestration of division, migration, and differentiation of multiple cell types, processes that depend on multiple growth factors and extracellular matrix components. Interference with any combination of these individual contributors can inhibit normal healing, emphasizing the extent to which healing requires the orchestrated interplay of many different components (including, for example, contributors as diverse as Wnt signaling, hair follicle development, β1 integrins, keratinocyte migration, extracellular matrix, macrophages, neovascularization, multiple growth factors, Schwann cells, appropriate TGF-β signaling, HIF-1 regulation, control of inflammation, etc. [1,2,32,50]. Despite the advances made in understanding the events required for effective skin wound healing, it has proven difficult to identify approaches that can enhance normal repair processes.

The ability to enhance normal wound healing with 4-AP is surprising for several reasons. 4-AP has the ability to enhance multiple processes associated with successful wound healing simultaneously. Our results are surprising because interfering with ion channel function seems less specific than more targeted approaches of cell transplantation or growth factor/cytokine manipulation during wound healing. Nonetheless, several calcium channel blockers also can enhance at least some aspects of the skin wound repair [10,12]. Only a subset of the outcomes in the present study was examined in these previous studies, which prevents a full comparison of the potency of 4-AP with these other calcium channel modulators. Nonetheless, these earlier studies do support the idea that modulating the function of at least some ion channels is a potentially useful approach to enhancing wound healing. That said, the benefits obtained with calcium channel blockers also raise concerns about whether 4-AP would be effective for this purpose, or whether it might even have adverse effects. This is because treatment with calcium channel blockers, such as verapamil, acts to inhibit calcium entry post-injury. In contrast, several studies on 4-AP predict that it might increase intracellular calcium levels [38,51,52]. Therefore, while it is intriguing that different classes of ion channel modulators can enhance the healing of normal skin wounds, the prior indications that 4-AP and calcium channel blockers might have opposite effects on intracellular calcium levels makes it difficult to offer a unifying hypothesis for these observations. Regardless of whether or not a common mechanism exists for the effects of these two classes of ion channel modulators, the data from our studies and previous studies on calcium channel blockers indicate that treatment with ion channel modulators offers a promising approach for enhancing the repair of skin wounds.

The ability of 4-AP to enhance regenerative processes post-injury was discovered only recently, and the present work provides still stronger support for the potential utility of this compound as a pro-regenerative agent than our previous findings. In our previous studies on peripheral nerve crush injuries, we found that 4-AP caused a more rapid return of motor function, a more rapid and greater return of nerve conduction, and increases in numbers of neurons and myelination [13]. The more rapid restoration of myelination and physiological function provides evidence for the pro-regenerative effects of 4-AP treatment. Some of the benefits observed in our previous studies could have been due, however, to the protective effects of this drug, as recently reported in models of CNS damage and multiple sclerosis patients [38,53]. However, in the case of full-excisional skin wound healing, outcomes such as those reported herein clearly require potential endogenous tissue regeneration.

The utilization of 4-AP to promote tissue repair is a qualitatively new use for this well-studied and clinically utilized drug. The positive tissue regenerative effects of 4-AP in the skin are very novel from the extensively studied ability of this drug to provide transient symptomatic relief, without evidence of regenerative changes, in chronic neurological illnesses and injuries such as multiple sclerosis, myasthenia gravis, cerebellar gait ataxias, downbeat and upbeat nystagmus, and spinal cord injury [54,55]. In neurological cases, the behavioral benefits of 4-AP are lost when treatment is terminated and the drug is cleared from the body. In contrast, the regenerative changes seen in acute traumatic injuries suggest that, in these settings, 4-AP treatment provides benefits that can endure long after treatment ends.

Whether any single target of 4-AP is most critical in skin wound healing, or whether modulation of multiple processes by this drug is critical, will be an interesting but challenging puzzle to solve. Wound healing processes are intricately connected and coordinated. We initiated the present experiments based on our previous findings that 4-AP treatment of acute peripheral nerve injuries enhanced SC and neuronal function post-injury and the importance of SC and neuron function in wound healing [1,5,13,56]. The multiple effects of 4-AP treatment, however, suggest that SCs and neurons were not the only components of successful wound healing impacted by this drug. For example, NGF, Substance P, and TGF-β levels were all elevated by 4-AP treatment, and each of these has been shown to have pro-reparative effects in skin wound lesions [1,7,29]. Substance P acts as mediator of wound healing by accelerating the inflammatory responses and enhances myofibroblast formation to produce collagen, keratinocytes migration, and re-epithelialization, but it is considered as a mediator of sensory symptoms such as pain, suggesting that possibility that pain responses may also be affected [46,48,49]. Our in vitro studies also indicated that 4-AP can have direct effects on keratinocyte migration and expression of SOX10 and NGF by these cells, indicating that the actions of 4-AP are not restricted to neuronal cell types. Consideration of direct molecular targets of 4-AP is also complex, includes multiple potassium and calcium channels, and may also include the ability to sequester intracellular calcium [57]. Skin cells, including keratinocytes, are dependent on Ca^2+^ signaling for proper differentiation programs further increasing the complexity of this problem. Elucidating the exact molecular mechanism(s) by which 4-AP enhances skin wound repair is challenging, but nonetheless, its translatability to the clinic is very promising. In contrast with the challenges in the mechanistic analysis of the effects of 4-AP, the translation of our findings to clinical studies is relatively straightforward. 4-AP fits the very definition of drug repositioning: using existing clinically useful compounds in novel applications. The ability to accelerate skin wound healing and skin regeneration is novel. Extensive prior studies on 4-AP safety and dosing [58], and its FDA approval in 2010 for the treatment of multiple sclerosis, make the transition of this compound from laboratory back to the clinical arena for wound healing therapy relatively straightforward. Indeed, our findings that 4-AP treatment can be used to distinguish between incomplete and complete peripheral nerve injuries have already transitioned to clinical trials on the diagnosis of such injuries (https://clinicaltrials.gov/ct2/show/NCT04026568; accessed on 1 July 2022) [13,15]. Moreover, our findings that 4-AP treatment enhances functional recovery from peripheral nerve damage have transitioned to clinical trials focused on enhancing recovery from peripheral nerve damage associated with radical prostatectomy, as manifested by urinary incontinence and erectile dysfunction (https://clinicaltrials.gov/ct2/show/NCT03701581; accessed on 1 July 2022) [13,15,56]. Thus, the possibility of bringing the present findings forward for clinical examination seems a promising one. In a broader sense, the large numbers of available drugs that modify ion channel function [59,60] offer multiple additional candidates of interest for their potential use in regenerative medicine.

## 5. Conclusions

The present study provides rationale for the novel therapeutic use and mechanism for 4-AP in cutaneous wound healing and tissue regeneration. 4-AP enhances numerous attributes of successful wound healing: re-epithelialization, dermal regeneration, and reinnervation. 4-AP may be acting on keratinocytes to upregulate transcriptional and neurotrophic factors. This work may provide a basis for the further study of 4-AP-mediated wound-healing effects by inducing nerve growth factor to promote tissue regeneration.

## Data Availability

The data presented in this study are available on request from the corresponding author.

## Supplementary Materials

The following supporting information can be downloaded at: https://www.mdpi.com/article/10.3390/biomedicines10071649/s1, Figure S1: 4-AP induced neuronal peptide wound healing and did not alter keratinocyte K10 expression; Figure S2: Characterization of human skin-derived primary keratinocytes and cell cytotoxicity assay with 4-AP; Video S1: An example time-lapse phase contrast images depicting the migration video of keratinocytes without treatment (control) during wound scratch closure. Images were recorded every one hour. Scale bar, 100 µm; Video S2: An example time-lapse phase contrast images depicting the migration video of keratinocytes after 4-AP treatment during wound scratch closure. Images were recorded every one hour. Scale bar, 100 µm.
