# Supplementary material for: 4-Aminopyridine Induces Nerve Growth Factor to Improve Skin Wound Healing and Tissue Regeneration
Source: Biomedicines. 2022 Jul 8;10(7):1649. doi: 10.3390/biomedicines10071649 (PMC9313269; doi:10.3390/biomedicines10071649)
Supplement: Supplementary file 1 [file biomedicines-10-01649-s001.zip › biomedicines-1786082-supplementary.pdf]

Supplementary information

4-aminopyridine induces nerve growth factor to improve skin wound healing and tissue regeneration

Mashanipalya G. Jagadeeshaprasad[1], Prem Kumar Govindappa[1], Amanda M. Nelson[2], Mark D. Noble[3] and John C. Elfar[1]

[1]Department of Orthopaedics and Rehabilitation, Center for Orthopaedic Research and Translational Science (CORTS), The Pennsylvania State University College of Medicine, Hershey, Pennsylvania, USA.

[2]Department of Dermatology, The Pennsylvania State University College of Medicine, Hershey, Pennsylvania, USA.

[3]Department of Biomedical Genetics, University of Rochester Stem Cell and Regenerative Medicine Institute, University of Rochester School of Medicine and Dentistry, Rochester, New York, USA.

Correspondence:

John C. Elfar, MD, FACS

Department of Orthopaedics and Rehabilitation

Center for Orthopaedic Research and Translational Science (CORTS)

The Pennsylvania State University College of Medicine and Milton S. Hershey Medical Center

Hershey, Pennsylvania 17033. Phone: 717-531-4686; Fax: 717-531-0349

E-mail: openelfar@gmail.com and jelfar@pennstatehealth.psu.edu

Supplementary figures and legends

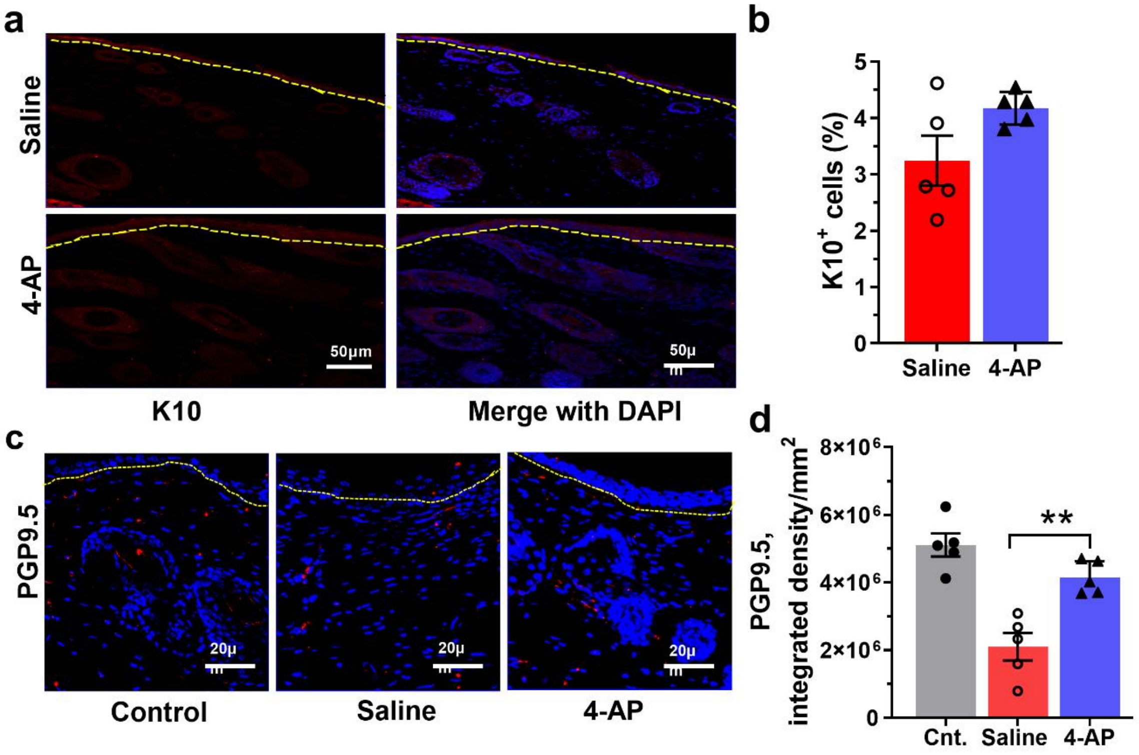

Supplementary Figure S1. 4-AP induced neuronal peptide wound healing and did not alter keratinocyte K10 expression. (a) Keratin 10 protein expression in the healed epidermis by immunofluorescence. 4-AP treatment did not cause any change in the expression of keratinocyte K10 expression. K10 (red); DAPI (blue) denotes nucleus and dashed line denotes epidermal/derma border. Scale bars, 50 µm. **(b)** Percent of $K10^+$ cells in control and 4-AP treated skin wounds at day 14. Each image represents 20 images from 5 different mice wound tissue and data represented as mean ± SEM, $n=5$ animals per group. **(c)** Immunofluorescence staining of control and healed wound sections for pan-neuronal marker PGP-9.5 (red) and nuclear stain DAPI (blue) denotes nucleus and dashed line denotes epidermal/derma border. Scale bars, 20 µm. **(d)** Quantification of PGP-9.5 protein-expressing cells showed significantly increased PGP-9.5 intensity in the 4-AP treated group compared to the saline-treated group on day 14. PGP 9.5 in 4-AP-treated mice was not significantly different from seen in uninjured (control) tissue. Each image represents 20 images from 5 different mouse wounds and data are represented as mean ± SEM, $n = 5$

animals per group, with statistical significance indicated by asterisks (** = P between 0.01 and 0.001 vs. saline).

Supplementary Figure S2. Characterization of human skin-derived primary keratinocytes and cell cytotoxicity assay with 4-AP. **(a)** Keratinocytes were characterized using keratin 14 (K14, a marker of proliferative keratinocytes) and K10 (keratin 10, a marker of keratinocyte differentiation marker). Scale bars = 100µm. **(b)** Cell viability using MTT assay with different concentrations of 4-AP (ranging from 1 to 10000 µM) for keratinocytes.

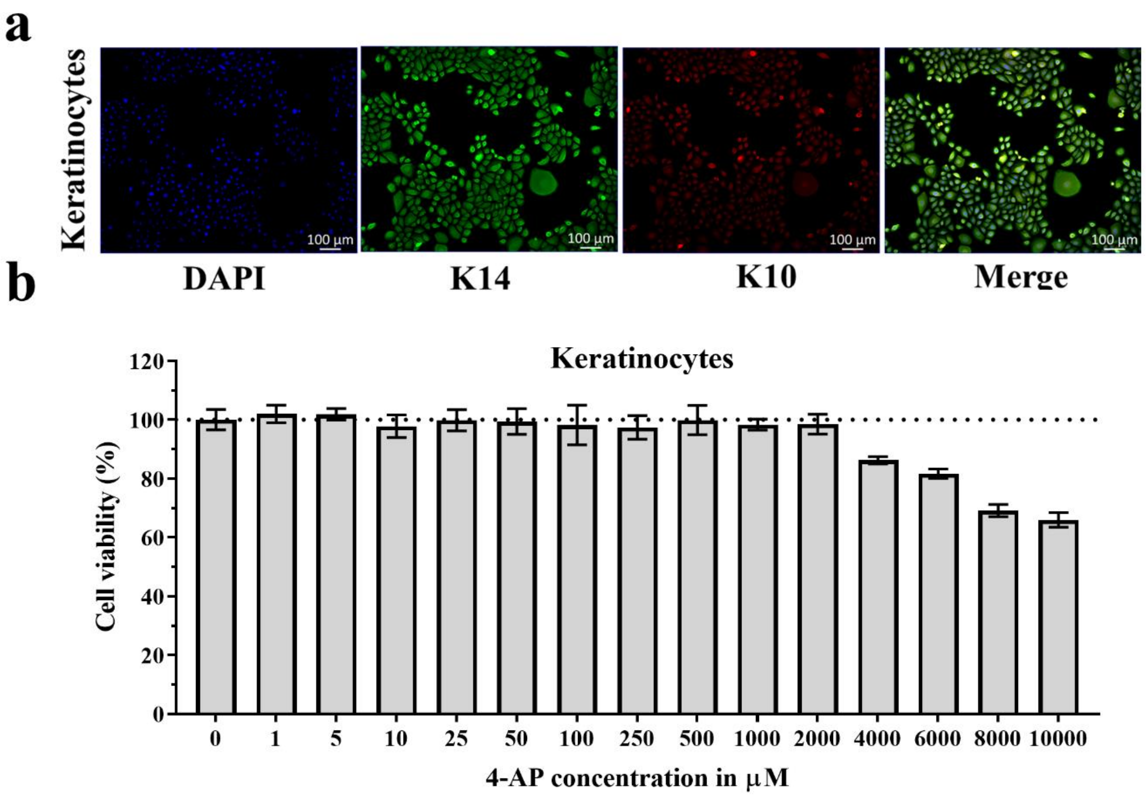

Supplementary movies

Supplementary Video S1. An example time-lapse phase contrast images depicting the migration video of keratinocytes without treatment (control) during wound scratch closure. Images were recorded every one hour. Scale bar, 100 µm.

Supplementary Video S2. An example time-lapse phase contrast images depicting the migration video of keratinocytes after 4-AP treatment during wound scratch closure. Images were recorded every one hour. Scale bar, 100 µm.
